# Uncertainties of healthcare professionals and informal caregivers in rare diseases: A systematic review

**DOI:** 10.1016/j.heliyon.2024.e38677

**Published:** 2024-09-28

**Authors:** David Zybarth, Laura Inhestern, Ramona Otto, Corinna Bergelt

**Affiliations:** a University Medical Center Hamburg-Eppendorf, Department of Medical Psychology, Germany; b University Medical Center Hamburg-Eppendorf, Health Services Research in Dermatology and Nursing, Germany; c University Medical Center Greifswald, Department of Medical Psychology, Germany

**Keywords:** Uncertainty, Rare diseases, Healthcare professionals, Informal caregiver

## Abstract

Uncertainties, defined as metacognitive awareness of ignorance, are an essential part of medicine. Consequently, healthcare professionals (HCPs) as well as informal caregivers face them inevitably. Depending on the interpretation of uncertainties and the existence of available resources to cope with them, uncertainties might have serious consequences. Studies showed higher burnout-rates and reduced psychosocial well-being of HCPs and informal caregivers. Especially rare diseases are linked with a variety of uncertainties, as knowledge about specific diseases is often limited which might result in a higher burden of both groups. This review aimed at summarizing studies dealing with HCPs' and informal caregivers’ uncertainties in the context of rare diseases. We searched five databases and screened 11.236 records for title/abstract and 105 records for full-text. Finally, 24 studies were subjected to quality assessment and data extraction using narrative synthesis. Five studies focused on HCPs, 19 on informal caregivers. Results were clustered using an existing taxonomy differentiating three categories of uncertainty (scientific, practical and personal) and issues, specifying particular uncertain situations or circumstances. Only five of the included studies investigated the perspective of HCPs, indicating a research gap of the topic within this group. Reports were mostly limited to practical and scientific uncertainties. Concerning scientific uncertainties, information procurement showed up as a special facet in rare diseases. Informal caregivers reported the whole variety of scientific, practical and personal uncertainties, leading to psychological consequences such as fear, confusion and worry. This review provides an overview of categories and assigned issues of uncertainties HCPs and informal caregivers experience in relation to rare diseases and psychological consequences. Results can be used in the development of trainings, to teach effective coping strategies when dealing with uncertainties or offers of psychosocial support.

## Highlights

• Uncertainties are an essential issue in medicine.
• Uncertainties of healthcare professionals are a neglected topic.
• “information procurement” is a unique uncertainty in rare diseases.
• Informal caregivers experience a variety of uncertainties.

## 1 Introduction

Deciding what to do when having a cold was no big deal for most people – at least before the beginning of 2020. With the onset of the COVID pandemic, new questions arose as people had to decide whether symptoms were caused by a typical cold or by the new virus. They were confronted with an essential part of medicine: uncertainty, which is defined as “the metacognitive awareness of ignorance” [1]. Although ignored most of the time, uncertainty affects nearly each part of clinical practice. Han et al. distinguish three dimensions of uncertainty: source, issue and locus [1]. *Source* describes the reason for uncertainty, for instance due to the probabilistic nature of a situation, or insufficient information. *Issues* describe specific situations the uncertainty occurs in. *Locus* refers to the subjective nature of uncertainties and highlights that patients and clinicians might perceive uncertain situations differently. Concerning issues of uncertainty, three main categories can be distinguished: scientific, practical, and personal uncertainties, with each category comprising more specific issues, such as making a diagnosis, deciding for an adequate treatment but also dealing with existential questions and psychosocial issues [1]. Scientific uncertainties are data or disease-centred, practical uncertainties system-centred and personal uncertainties patient-centred. The taxonomy has been used by various studies and modified to suit specific contexts (e.g. Pomare et al. [2]). An illustration of the taxonomy [1] is provided in Table 1. As healthcare professionals (HCPs) and, in the case of children, informal caregivers are responsible for their patients or relatives, surrogate decision-making is an established practice in the medical context [3] and might be challenging for the deciding person, as individuals differ in their tolerance for uncertainty. Not addressing the ability to tolerate uncertainty has important implications for clinical practice. Based on the transactional stress model by Lazarus and Folkman [4] individuals primarily appraise external stressors/factors (e.g., uncertain situations) as positive, dangerous, or irrelevant. If classified as dangerous, these stressors lead to a secondary appraisal of the available resources. Should these be insufficient and coping strategies are not successful, individuals experience stress that might result in reduced well-being if coping strategies are not successful [5]. Due to its generalizability, the transactional stress model is used in a variety of contexts. It is therefore also suitable for the characterization of uncertainty. In line with the model, uncertainties can be described as stressors that may lead to a dysfunctional secondary appraisal. Distinguishing between stressors and appraisal is another important aspect for choosing the transactional stress model [4]. Although HCPs in a certain medical field experience similar uncertainties not all of them will be negatively affected by them. The effect depends on an individual's uncertainty tolerance. In fact, lower uncertainty tolerance impairs the accessibility of available resources, resulting in anxiety, depressive symptoms or reduced subjective well-being in caregivers [6,7]. Specifically in HCPs, lower uncertainty tolerance is associated with higher burnout rates, lower job satisfaction, and reduced mental well-being [[8], [9], [10]]. Despite these consequences and its omnipresence in clinical situations, the topic is heavily underrepresented in research. One of the reasons might be the conjunction of uncertainty with “fear of personal inadequacy and failure”, already described by Gerrity et al., in 1992 [11]. HCPs may tend to avoid this categorisation and therefore order multiple, sometimes unnecessary, tests that lead to increased healthcare costs [12,13].

**Table 1:** Issues of uncertainty, adapted from Han et al. (2011).

Category of uncertainty	Issue of uncertainty	Guiding question to assign uncertainty
Scientific uncertainty	Diagnosis	What uncertainties do HCPs or caregivers encounter when searching for a diagnosis?
	Prognosis	What uncertainties do HCPs or caregivers experience in predicting the course of the disease?
	Causal explanations	What uncertainties do HCPs or caregivers have in the causal explanation of the cause of illness?
	Treatment recommendations	What uncertainties do HCPs or caregivers encounter when treating a diagnosed disease?
Practical uncertainty	Structures of care	What uncertainties do HCPs or caregivers have about structures of care and responsibilities of particular individuals or institutions?
	Processes of care	What uncertainties do HCPs or caregivers have about (logistical) issues of organizing or paying for care, including necessary actions a person must take to access care.
Personal uncertainty	Psychosocial	What uncertainties are triggered by the disease in interpersonal relationships?
	Existential	What uncertainties does the disease cause in terms of life planning and quality of life?

When dealing with rare diseases, the number of possible issues of uncertainty rises enormously as knowledge in this context is limited [14]. In the European Union, a disease is defined as rare when it affects less than 5 in 10.000 people [15]. The estimated 5.000–8.000 different rare diseases collectively affect 27 to 36 million individuals in the European Union [16]. The rarity poses special challenges because variability between different diseases is high, time to diagnosis is often longer than for common conditions, and treatment options are available for only 5 % of rare diseases. The term *diagnostic odyssey* describes the long journey of misdiagnoses patients with a rare disease frequently experience [17]. This odyssey poses an additional burden for affected individuals, that impacts psychological wellbeing and health related quality of life [18,19]. But also an economic burden for the society as a whole [13,20]. Since 50 % of all rare diseases start in childhood, this poses an extra burden on informal caregivers, who in most cases are the parents [21]. Besides emotional stress, parents also have to handle consequences from care work such as negative impacts on careers and reduced income [22].

It remains unclear whether the uncertainties HCPs and informal caregivers experience in the context of rare diseases are comparable to other diseases or whether they pose a unique challenge. A further open question is whether both groups, HCPs and informal caregivers, experience similar uncertainties. As discussed above, both groups are in the special position of surrogate decision-making [3]. Assuming similarities raises the question, if resulting effects are comparable. Against this background, this review addresses these questions by identifying studies dealing with uncertainties of HCPs and informal caregivers caring for patients with rare diseases or suspected rare diseases. It intends to give an overview of 1) the uncertainties experienced by the HCPs and caregivers and 2) the psychological aspects (e.g. emotional, cognitive) associated with uncertainty. The results can be used in the development of trainings to support HCPs and informal caregivers in dealing with uncertainty and, hence, to improve care and health related quality of life for people affected by rare diseases.

## 2 Methods

This systematic review was conducted using the Preferred Reporting Items for Systematic Reviews (PRISMA) statement [23]. It was also registered with PROSPERO, registration code CRD42021219092.

### 2.1 Data sources and search strategy (including inclusion and exclusion criteria)

A systematic literature search was conducted in January 2021 using the following databases: APA PsycArticles, APA PsycInfo and PSYNDEXplus Databases via OVID platform, PubMed and CINAHL. Additionally, reference lists of included papers were hand-searched to identify relevant studies. Search terms represented the three domains: uncertainty, persons of interest and rare diseases, and were a mix of keywords and MeSH terms (supplementary material). In September 2023, an update was conducted using the same search terms. All types of peer-reviewed studies were included (qualitative, quantitative, mixed-methods). Full-texts had to be accessible, in English or German language and substantially focus on uncertainties experienced by HCPs or informal caregivers caring for people with rare diseases or suspected rare diseases (reasonable suspicion by HCP). Informal caregivers were limited to parental caregivers, regardless of the age of the child. No publication date restrictions were applied. Studies were excluded if they did not focus (at least partially) on uncertainties. To keep focus on groups responsible for surrogate decision-making (HCPs and parental caregivers) studies were excluded, if they focused on uncertainties experienced by patients with rare diseases themselves, if they focused on other informal caregiver groups (e.g. spouses, siblings), and if they focused on medically unexplained symptoms. Furthermore, reviews, meta-analyses, books and non-research publications (e.g. conference abstracts, commentaries) were excluded.

### 2.2 Study selection

First, one researcher (DZ) screened all titles and abstracts of the studies discovered through the search and additional resources for possible inclusion. A second researcher (RO) screened 10 % of randomly picked titles and abstracts to assure adequate quality and reliability. Second, both reviewers screened the full texts of the identified studies for eligibility applying the inclusion and exclusion criteria set out above. Any disagreements were discussed and resolved through consensus. Where necessary, a third reviewer (LI) was consulted. All decisions were documented using Microsoft Excel (Version 2019).

### 2.3 Data extraction

Data extraction was also documented, also using Microsoft Excel (Version 2019) and included the following characteristics: title, authors, year of publication, journal, type of uncertainties described, psychological aspects of experienced uncertainties (if reported), population, and sample size. Depending on their methodological approach, included studies were classified in quantitative design studies, qualitative design studies or mixed-methods studies. Studies were also classified by the target group: healthcare professionals or caregivers. Data extraction was performed by DZ and reviewed by RO independently. Disagreements were discussed and resolved through consensus.

### 2.4 Quality assessment

The quality of included studies was assessed by using the Mixed Method Appraisal Tool (MMAT), which is a widely used tool to assess quality in reviews including studies on different methodological approaches [24]. Two reviewers (DZ, RO) rated eligible studies independently. Disagreements were discussed and resolved through consensus.

### 2.5 Analyses

We used narrative summaries to synthesise the data from included studies [25]. This interpretative approach allows combining qualitative and quantitative data. As we did not expect a large amount of homogenous quantitative studies, meta-analysis was determined as not appropriate. Extraction of qualitative information was limited to the results sections of articles, excluding information that only derived from quotes and were not addressed otherwise by the authors. Uncertainties had to be stated explicitly, either by being called “uncertainties” or by corresponding to the given definition of uncertainties. The naming of the reported uncertainties corresponds to the naming in the included studies. In individual cases, they were paraphrased to improve readability and comparability between studies. After collection, uncertainties were deductively clustered to answer the research questions. Following the framework by Han et al. [1], each identified uncertainty was assigned to a specific issue of uncertainty, which in turn was assigned to one of the previously mentioned main categories of scientific, practical, and personal uncertainties. The framework is well established and has been used in other medical contexts or target groups. It therefore allows to compare the situation of HCPs and parents in the field of rare diseases with other contexts and groups.

## 3 Results

### 3.1 Included studies

**Fig. 1:**
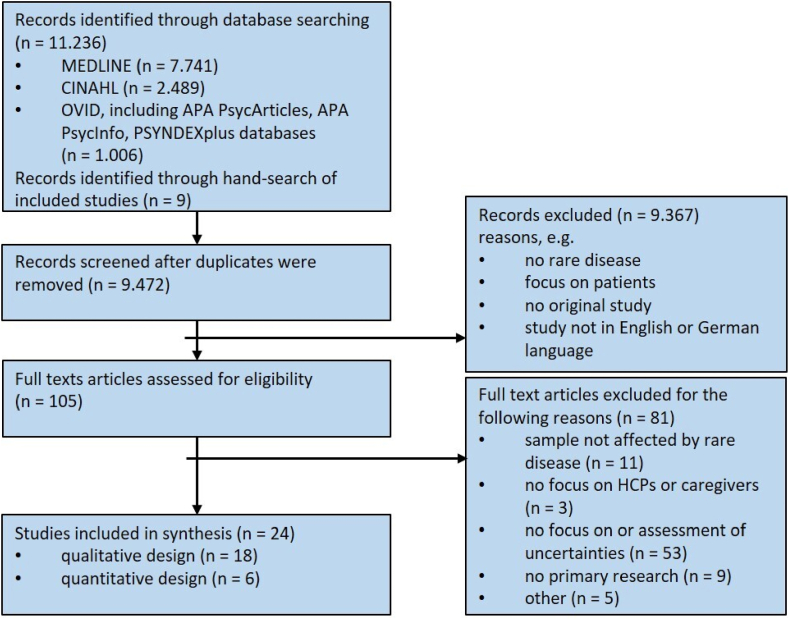
PRISMA flow diagram illustrating the study selection process.

Database- and hand-search resulted in 11.236 records, including 1.764 duplicates. After reviewing the remaining 9.472 studies, 9.367 were excluded based on screening of title and abstract. 105 studies were eligible for full text screening which resulted in a final number of 24 studies included in the narrative syntheses. After full text screening most studies were excluded because they did not assess uncertainties or did not focus on a sample, that was certainly affected by a rare disease (but e.g. on people who are carriers of a certain mutation without any symptoms). Fig. 1 illustrates the study selection process.

### 3.2 Health care professionals

We included five studies specifying uncertainties reported by HCPs. For study characteristics, see Table 2. Results are clustered in Table 3. Three studies used a quantitative approach, two a qualitative approach. No study focused on a specific rare disease, all investigated uncertainties associated with rare diseases in general or with a defined spectrum of diseases. One study defined the term “uncertainty” explicitly [26] but all used it in line with the mentioned framework. Sample sizes ranged between 169 and 242 for quantitative studies. One study only included paediatricians [27]; the remaining four studies included HCPs in general. Two studies were conducted in Europe, two in Canada and one in Australia. Most prominent issues belong to the category of practical uncertainties, namely *availability of peer* support, *patient referral* and *clinical pathways* [[27], [28], [29]]. In contrast to the existing frameworks, *information procurement* emerged as a new issue of scientific uncertainty [29].

**Table 2:** Characteristics of included studies related to HCPs (n = 5).

Study, country and method	Study aim/purpose	Participants	Type of rare disease	Uncertain issues	Psychosocial consequences
Azzopardi, Upshur et al. (2020) Canada qualitative interview study	navigation of medical uncertainty	HCPs (n = 12)	atypical forms of inherited metabolic diseases	diagnosis, treatment, prognosis of rare diseases, communication with parents, potential harm of diagnosis without evidence about treatment	none reported
Nguyen and Charlebois (2015) Canada qualitative interview study	perspective of HCPs on whole-exome sequencing	HCPs (n = 10)	rare diseases in general	decision for adequate genetic test, whole-genome sequencing: interpretation of results, differentiation between relevant and irrelevant results	none reported
Ramalle-Gómara, Domínguez-Garrido et al. (2020) Spain quantitative online-questionnaire survey	training needs and perceived shortcomings of HCPs	HCPs (n = 169)	rare diseases in general	where to refer patients to (62.2 % of hospital HCPs, 66.7 % of primary care HCPs), procedure of patient referral, availability of peer support for patients and families (37.8 % of hospital HCPs, 44.4 % of primary care HCPs)	none reported
Vandeborne, van Overbeeke et al. (2019) Belgium quantitative online-questionnaire survey	Information and education needs of HCPs	HCPs (n = 295)	rare diseases in general	specific sources of information about rare diseases and ultra-rare diseases, prevention and screening of rare diseases, patient referral, differential diagnosis, rare disease symptoms, orphan drugs, treatment of rare diseases	none reported
Zurynski, Gonzalez et al. (2017) Australia quantitative online-questionnaire survey	difficulties, information-seeking behaviours, educational needs and preferences of paediatricians	paediatricians (n = 242)	rare diseases in general	availability of peer-support groups for patients and families (35 %), patient referral (21 %)	none reported

HCP = healthcare professional.

**Table 3:** Uncertainties of HCPs identified by included studies (n = 5).

Category	Issues	Reported uncertainties
Scientific	Diagnosis^1^	Adequate genetic test/interpretation of results ^2^ Prevention and screening of rare diseases ^4^ Differential diagnosis/rare disease symptoms ^4^
	Prognosis	Prognosis in general ^1^
	Treatment (recommendations) ^1,4^	Orphan drugs ^4^
	Information procurement∗	Specific sources of information about rare and ultra-rare diseases^4^
Practical	Structure of care	Patient referral/clinical pathways ^3,4,5^ Availability of peer support ^3,5^
	Process of care	Procedure of patient referral ^3^
Personal	Psychosocial	Communication with parents ^1^
	Ethical∗∗	Potential harm of diagnoses without evidence of treatment ^1^

∗New issue, not part of existing taxonomies; ∗∗issue added by Pomare et al. [2]; ^1^Azzopardi, Upshur et al. (2020), ^2^Nguyen and Charlebois (2015), ^3^Ramalle-Gómara, Domínguez-Garrido et al. (2020), ^4^Vandeborne, van Overbeeke et al. (2019), ^5^Zurynski, Gonzalez et al. (2017).

### 3.3 Informal caregivers

Nineteen studies specified uncertainties of informal caregivers. Parents reported all categories of uncertainties, focussing on scientific and practical uncertainties with a variety of specific uncertainties within each category. Study characteristics are presented in Table 4, clustered results in Table 5. Most identified studies (n = 17) used a qualitative approach. Two used a quantitative design. Most studies focused on a specific rare disease (n = 13), while four studies included a defined spectrum of diseases. Less than half of studies (n = 8) defined or explained the term “uncertainty” [[30], [31], [32], [33], [34], [35], [36], [37]] but again all used it in line with the framework developed by Han et al. [1]. The study participants mainly or exclusively were mothers in all studies, except for one study that included mothers and fathers equally [36]. Four studies reported having included parents without specifying gender ratios [34,[37], [38], [39]]. Sample sizes ranged between 8 and 132 for qualitative studies and between 178 and 363 in quantitative studies. One study used observations of physician-parent-child interactions as an additional source of information [31]. Most studies were conducted in the USA (n = 11). The remaining studies were conducted in the Netherlands (n = 3), Canada (n = 2), the United Kingdom (n = 1) and Australia (n = 1). One study was a collaboration project between researchers from the United Kingdom and Sweden.

**Table 4:** Characteristics of included studies related to informal caregivers (n = 19).

Study, country and method	Study aim/purpose	Participants	Type of rare disease	Uncertain issues	Psychosocial consequences
de Ru, Bouwman et al. (2012) Netherlands qualitative interview study	experiences of patients and parents with timing of diagnosis	parents (n = 13) and teenage/adult patients (n = 6)	Mucopoly-saccharidosis type I	diagnostic odyssey (child showing symptoms without diagnosis)	feelings of powerlessness, frustration, impotence and distress
Faux, Schoch et al. (2012) USA qualitative interview study	factors related to disclosing a diagnosis	mothers (n = 8)	22q11.2 deletion syndrome	disclosure of diagnosis to child	none reported
Hamilton, Hutson et al. (2013) USA quantitative online-questionnaire survey	association between uncertainty and decision-making	parents (n = 178; n = 109 female)	Faconi anemia	conflicting scientific evidence about stem cell transplantation (M = 2,47, SD = 0,95, scale 1–4) and lack of information about transplant (M = 1,64, SD = 0,89, scale 1–4), no significant associations; probabilistic effectiveness of transplant (M = 2,21, SD = 1,07, scale 1–4), significant influence on likelihood of choosing a transplant; conflicting expert opinions about transplant (M = 2,01, SD = 1,01, scale 1–4), significant influence on decision-making difficulties about undergoing stem cell transplantation	conflicting expert opinions associated with greater decision-making difficulties
Hinton and Armstrong (2020) United Kingdom qualitative interview study	navigation of uncertainty associated with neonatal surgery	Parents (n = 42, n = 31 female)	rare congenital conditions	future treatment; prognosis; everyday care for child	anxiety and feelings of disempowerment
Inglese, Elliott et al. (2019) Canada qualitative interview study	exploration of family experiences related to new syndromes	parents (n = 11; n = 8 female), sibling (n = 1)	any newly identified syndromes	child's health and future, life expectancy	fear, anxiety, loss of control
Kalbfell, Wang et al. (2023) USA qualitative interview study	disease related burdens of young adult patients and parents	parents (n = 34, n = 28 female), patients (n = 25, n = 21 female, n = 2 male)	vascular malformations	diagnostic odyssey, limited treatment options, child's future (health)	none reported
Kerr and Haas (2014) USA qualitative observations of physician-parent-child interactions, parents discussions and questionnaire with open ended questions	exploration of uncertainties experiences by parents of children with rare diseases	questionnaire: parents (n = 55; n = 46 mothers); observations: child-parents-physician interaction (n = 200)	rare vascular anomalies	“Normalization Uncertainty”: child's (social) future, child's ability to be “normal”, uncertainty regarding parents' future reproduction plans; “Information Uncertainty”: inadequate (treatment) information especially regarding surgery of birthmarks; “Orphan-Illness Uncertainty”: influence of birthmarks on other parts of the body, uncertainty about complex health risks; “Parental Proxy Uncertainty”: assessment of pain and decision when to seek care; “Social Stigma Uncertainty"	none reported
Kutsa, Andrews et al. (2022) USA qualitative interview study	exploration of disease related uncertainties and coping	parents (n = 26, n = 21 female)	severe combined immuno-deficiency	understanding the diagnosis, treatment options, medical insurance and financial resources, care for other family members, prognosis, child's future (health), life expectancy	feelings of confusion, anxiety, sadness, depression, worry, guilt; neglection of own well-being
Lipinski, Lipinski et al. (2006) USA quantitative online/paper-pencil questionnaire	association between parental uncertainty/perceived control and perceived helpfulness of a genetic counselor	parents (n = 363; n = 346 female)	rare chromosome disorders	mean uncertainty score: 82,7 (SD = 14,5; PPUS, range 31–155); negatively correlated with uncertainty score: perceived benefit of diagnosis (−0,25), perceived helpfulness of genetic counselor (−0,2), perceived personal control (−0.32), parental age (−0,13); positively correlated with uncertainty: perceived seriousness of condition (0,17)	none reported
Lundberg, Lindström et al. (2017) United Kingdom, Sweden qualitative interview study	investigation of different kinds of knowing parents use when learning how to care for their child	parents (n = 20)	Congenital Adrenal Hyperplasia	missing diagnosis in the presence of symptoms; understanding the diagnosis; gender assignment; dealing with new situation; talking to others about diagnosis; knowledge about medication; how to administer medication; seeking emergency care; communication with child about disease/surgery; independence of child	confusion, loneliness after diagnosis due to partial or too much information; feeling of insecurity when confronted with new situation
Pruniski, Lisi and Ali (2018) USA qualitative interview study	effects of diagnosis via newborn screening on parents and families	mothers (n = 9)	Pompe disease	late onset Pompe disease: verification and understanding of diagnosis, symptom onset, treatment initiation, child's future; infant onset Pompe disease: additionally, uncertainty about ensuring current child health	anxiety and fear
Qian, McGraw et al. (2015) USA focus groups, qualitative interview study	disease related experiences of patients with spinal muscular atrophy and their families	parents (n = 64; n = 49 female), HCPs (n = 11), patients (n = 21)	Spinal Muscular Atrophy	development of disease, life expectancy	feeling of helplessness and loss of control
Raspa, Kutsa et al. (2023) USA qualitative interview study	understanding different types of uncertainty experienced by parents of children with severe combined immunodeficiency	parents (n = 26, n = 21 female)	severe combined immuno-deficiency	validity of newborn screening result, understanding the diagnosis, causality of genetic pathways, treatment decisions, prognosis, possible complications of treatment, parental jobs and careers, costs of treatment, child's future (health), household management, long-term treatment, relationships (within the family, with extended family and friends), life expectancy, long-term effects of decisions, effects of COVID-19 on well-being	anxiety, worry, fear, feelings of doubt (about screening results), confusion, impression of overwhelming demands, guilt, grief, anger, frustration, depression
Smits, Vissers et al. (2022) Netherlands qualitative interview study	exploration of the experience of having a child with a rare disease	parents (n = 12)	rare diseases in general	diagnostic odyssey, treatment decisions, child's future (health)	distress, anxiety
Tluczek, McKechnie and Lynam (2010) USA qualitative interview study	examination of psychosocial consequences of newborn screening in case of ambiguous results	parents (n = 10; n = 5 female)	Cystic fibrosis	contradictory test results; prognosis; distinguish normal childhood problems from Cystic fibrosis' symptoms	change of affective responses over time, less intense distress; initial feelings of sadness, anger, guilt, blame, somatic symptoms; worrying; feeling isolated
van Scheppingen, Lettinga et al. (2008) Netherlands qualitative interview study	identification and specification of problems of parents with a child suffering from epidermolysis bullosa	parents (n = 17; n = 11 female; n = 6 couples)	Epidermolysis bullosa	parents of mildly and severely affected children: unpredictability of child's illness, prognosis; parents of severely affected children: life expectancy	feelings of guilt
Whitmarsh, Davis et al. (2007) USA qualitative interview study	interpretation of confirmed genetic diagnoses by parents and grandparents	parents (n = 132; n = 122 female), grandmother (n = 1)	Turner, Fragile X (only part of analyses, recruited in another study), Klinefelder (not rare)	indefiniteness of syndrome, prognosis, variation of symptoms (unclear symptom onset)	frustration (only if the genetic diagnosis is perceived as certain)
Withers, Fleming et al. (2020) Australia qualitative interview study	disease related experiences of parents	parents (n = 13, n = 10 female)	Rubinstein-Taybi syndrome	child's future health	none reported
Xiao, Kang et al. (2023) Canada qualitative interview study	experiences of caregivers with disease-modifying therapies of spinal muscular atrophy	parents (n = 15, n = 11 female)	Spinal muscular atrophy	missing long term safety and efficacy data of medication; future provision and funding of medication	fear of future health impairments of the child

M = mean, SD = standard deviation, HCP = healthcare professional, PPUS=Parents' Perception of Uncertainty Scale.

**Table 5:** Uncertainties of informal caregivers identified by included studies (n = 19).

Category	Issues	Reported uncertainties
Scientific	Diagnosis	Diagnostic odyssey ^1,6,10,14^ Understanding of diagnosis ^8,10,11,13^ Verification of diagnosis (e.g. due to contradictory test results) ^11,13,15^ Differentiation between rare disease symptoms and common (disease) symptoms ^15^
	Prognosis ^4,5,6,8,12,13,14,15,16,18^	Indefiniteness of syndrome, unpredictability of disease and symptoms ^11,16,17^ Complex health risks associated with disease ^7^ Life expectancy ^5,8,12,13,16^ Future treatment ^4^
	Causal explanations	Causality of genetic pathways ^13^
	Treatment (recommendations)	Conflicting expert opinions ^3^ Effectiveness of treatment ^3^ Possible complications of treatment/missing long-term safety and efficacy ^13,19^ Lack of information/knowledge about treatment ^7,10^ Timing of treatment initiation ^11^ Limited treatment options ^6^ Understanding treatment decisions/options ^8,14^ Deciding for a treatment ^13^
Practical	Structures of care	(Future) provision and funding of treatment ^8,13,19^ Clinical responsibility (e.g. confirmation of diagnosis) ^13^
	Processes of care	Ensuring current health by appropriate examinations ^11^ Assessment of pain ^7^ Deciding when to seek emergency care ^7,10^ How to administer medication ^10^ Ensuring everyday care for child ^4^ Ensuring care of other family members ^8^ Household management ^13^
Personal	Psychosocial	Communication with child about disease or treatment/disclosure of diagnosis ^2,10^ Talking to others about diagnosis ^10^ Effects on relationships to family, extended family, friends ^13^ Effects of current decisions on the later relationship with child ^13^
	Existential	Independence of child ^10^ Child's ability to be “normal” (e.g. participation in school activities) ^7^ Social stigma ^7^ Child's future (e.g. marriage, children, job, insurance) ^5,8,11,14^ Dealing with new situation of having a child with a rare disease ^10^ Effects of disease on parental jobs and careers ^13^ Parental reproduction plans ^7^ Health and well-being due to COVID-19 ^13^

^1^de Ru, Bouwman et al. (2012), ^2^Faux, Schoch et al. (2012), ^3^Hamilton, Hutson et al. (2013), ^4^Hinton and Armstrong (2020), ^5^Inglese, Elliott et al. (2019), ^6^Kalbfell, Wang et al. (2023), ^7^Kerr and Haas (2014), ^8^Kutsa, Andrews et al. (2022), ^9^ Lipinski, Lipinski et al. (2006), ^10^Lundberg, Lindström et al. (2017), ^11^Pruniski, Lisi and Ali (2018), ^12^Qian, McGraw et al. (2015), ^13^Raspa, Kutsa et al. (2023), ^14^Smits, Vissers et al. (2022), ^15^Tluczek, McKechnie and Lynam (2010), ^16^van Scheppingen, Lettinga et al. (2008), ^17^Whitmarsh, Davis et al. (2007), ^18^Withers, Fleming et al. (2020), ^19^Xiao, Kang et al. (2023).

## 4 Discussion

This systematic review provides an overview of studies dealing with uncertainties of HCPs and informal caregivers in the context of rare diseases. Only five studies were identified that investigated uncertainties of HCPs in the context of rare diseases, reflecting a research gap within this group. Included studies focus on scientific and practical uncertainties, mostly neglecting personal uncertainties. Only Azzopardi et al. [26] report personal psychosocial and ethical uncertainties by questioning the benefits of *diagnoses without evident treatment options* and difficulties in *talking to parents*. One reason for the focus on scientific and practical uncertainties could be that most studies used self-designed online surveys that ask about predefined uncertainties, not providing the opportunity to articulate personal experiences. Consequently, results are largely limited to medical aspects of care. Again, only Azzopardi et al. [19] chose a qualitative approach. As our synthesis shows, *diagnosing* and *treating rare diseases* are relevant uncertainties according to HCPs [29,40]. Especially *differentiating rare diseases from common ones* is a challenge [29]. Making this problem more complex, 73 % of general practitioners have *never heard of any of the relevant organizations, search engines or information sources relevant in the field of rare diseases*, compared to only 14 % of paediatricians [29]. Besides knowing information sources about rare diseases, a significant number of general practitioners (22 %), but also paediatricians (15 %) and adult specialists (15 %) does not know, where to find correct information about rare diseases at all [29]. In contrast to common, even complex diseases, this opens a new issue of scientific uncertainties in the field of rare diseases which existing classifications do not cover. The uncertainty is referred to as *information procurement* in this review. Instead of questioning existing information, HCPs do not know valid sources of information and do not have established contacts to get the information needed. Furthermore, up to 67 % of primary care physicians admit to lack knowledge about *patient referral* to and up to 44 % are uncertain about *availability of peer-*support for patients and families [28]. In comparison, the study examined by Zurynski et al. [27] that only included paediatricians, showed lower levels in uncertainties about *patient referral* (21 %) and *availability of peer-*support (35 %). Concerning rare diseases, this might indicate differences in academic medical education, work experiences and/or continuous training between paediatricians and HCPs working with adults. Most rare diseases manifest in childhood necessitating paediatricians to have a more profound knowledge of rare diseases [41]. Few studies investigated differences between medical specialities. As far as the uncertainties are concerned, no further differences were identified apart from those already mentioned [28,29]. To overcome educational deficits Walkowiak and Domaradzki [42] suggest to implement rare diseases as a mandatory topic in university curricula and to involve patient organizations in raising awareness for patients' problems. However, uncertainty about *medical pathways* is highly problematic as the consequences of patients’ diagnostic odysseys and missed therapeutic windows of diseases might be serious [43]. Considering a rate of 80 % genetically determined rare diseases, genetic testing seems to be a promising solution to speed up the diagnostic process and to cope with uncertainties [21]. In fact, genetic testing, just like other innovations, entails new uncertainties such as *deciding for an appropriate test* when a rare disease is suspected or *interpreting results* after genetic testing was performed [40]. Artificial intelligence provides promising solutions to overcome these uncertainties. Algorithms can support the diagnostic process by identifying symptom patterns that HCPs might not detect [44]. An interview study of German HCPs shows that especially for rare diseases participants hope for a better diagnostic quality through the use of artificial intelligence [45]. Additionally, artificial intelligence has the potential to improve the treatment planning through predictive modelling techniques and the support of orphan drug development [44,46]. Besides all promises, rare diseases pose special challenges to artificial intelligence as well. Due to small number of cases per disease, the availability of data is limited which can lead to biased results of models [46]. To overcome these shortcomings and use the whole potential of artificial intelligence, the technology needs to be embedded in a suitable framework as described by Bragazzi and Garbarino [47]. Besides the general focus on scientific and practical uncertainties, no study reported psychosocial consequences of uncertainties in HCPs.

Nineteen studies were identified for informal caregivers, indicating a much greater acceptance of assessing uncertainties in this group. Nevertheless, only a minority of authors defined or explained the construct of uncertainty explicitly. In contrast to studies including HCPs, here most studies focused on specific diseases or groups of diseases and used a qualitative approach, which allowed a wider coverage of uncertainties. While HCPs and informal caregivers share some aspects of scientific uncertainties, such as *confirmation of suspected diagnosis* or (*initiation of) appropriate treatments* [30,31,48], informal caregivers' uncertainties also arise from the necessity to *understand the diagnosis or treatment options* [32,34,35,48]. Understanding the disease and making proper treatment decisions is an important step in answering prognostic questions which are a major uncertainty for parents as they may affect a *limited life expectancy* of their child [[49], [50], [51]] and an *unpredictable development of the disease* [32,35,37,[48], [49], [50],^52^]. These uncertainties lead to feelings of losing control, guilt, fear, anxiety, grief and depression [32,35,49]. They are frequently accompanied and even intensified by *limited treatment options* [53] and other treatment uncertainties such as *missing long-term data about safety and efficacy* of treatments [54] or possible *(future) complications of treatments* [35], leading to fear of future health impairments of the child [54]. Although prognostic and treatment uncertainties are part of any disease, they take on a special role against the background of limited information in the context of rare diseases, which, as mentioned above, naturally also affects HCPs. Especially in non-metropolitan areas or non-specialized healthcare settings parents perceive a lack of understanding and knowledge of HCPs [55,56] which might contribute to existing uncertainties and strain the caregiver-HCP relationship [57]. A disease-specific healthcare setting on the other hand seems to positively influence caregiver's satisfaction [58]. A mixed methods study investigating patient and caregiver satisfaction with centres for rare diseases confirmed the positive influence and found that most participants were satisfied with the centres, felt understood by the professionals at the centres and had confidence in the treatments [59]. The same study also analysed the collaboration between local HCPs and centres for rare diseases. To raise awareness of rare diseases in general and improve the situation for patients and caregivers, cooperation between stakeholders is essential. Anyway, patients and caregivers reported delays in the transmission of information between stakeholders, making them responsible for organizing intersectoral collaboration [59]. This puts an additional burden on caregivers and patients [60]. To improve the collaboration a number of suggestions were made [61]. Besides efforts to implement electronic patient files [62], patient organizations play an important role to navigate patients and caregivers through the healthcare system and to provide them with the information, their local HCP might lack [59]. Additionally, patient organizations, as well as online groups and communities are an important source of support for patients and caregivers [63,64].

Caregivers also experience practical uncertainties related to treatment, such as *difficulties in administering medication* [34]. Due to *unpredictable symptoms*, which can be difficult to *distinguish from common problems* [36] and make it difficult *deciding, when to seek (emergency) care* [31,34], caregivers might experience feelings of isolation, loneliness, confusion or worry [34,36]. All consequences indicate an intense stress reaction, a risk factor for reduced mental well-being and social functioning in the long run [65]. Practical uncertainties, such as *household management* [35], the *everyday care of the ill child* [66] or *care of another (healthy) family member* [32] further intensify the stress reaction and might result in feelings of disempowerment [66]. One included study revealed that higher uncertainty was associated with lower perceived control [33]. In the transactional stress model [4], the perceived control affects a person's stress response, with lower control leading to higher stress, which can impact psychological well-being [5].

Apart from existential issues directly addressing the *child's future* [32,35,39,48,49,53] or current *ability to live a “normal” life* [31], parents also face multiple facets of personal uncertainty in other aspects of life. By questioning or changing *reproduction plans*, parents experience extensive existential uncertainties [31]. These are accompanied by uncertainties regarding *job/career perspectives* [35] and psychosocial uncertainties of *how to talk to the child or other people about the disease* [34,67]. Again, following the transactional stress model these situations pose relevant stressors to parents that might exceed their coping capabilities.

## 5 Limitations

This review provides an overview of uncertainties experienced by HCPs and informal caregivers in the context of rare diseases. Although one of the core topics in each article, most authors did not define the construct of uncertainty. Due to the lack of theoretical background, it was difficult to assess the exact significance of the uncertainties mentioned and to clearly assign them to the framework used here. As Han et al. [1] stated, uncertainties might be interconnected and one statement can be attributed to different issues. Nevertheless, we assigned each uncertainty to only one issue. Furthermore, the limited range of identified uncertainties might be due to a methodological bias of some studies. Another problem is the conflation of “uncertainty” with related concepts such as “information needs”, in case of HCPs or “worry”, in case of informal caregivers.

## 6 Conclusion

Uncertainty is a complex construct that poses challenges in multiple areas of caregiving in the medical context. Depending on their uncertainty tolerance, HCPs and informal caregivers might be overwhelmed by these challenges, resulting in a heightened psychosocial burden [9,38,49]. For parents, multiple studies support this conclusion. In contrast, only one study dealt with personal uncertainties of HCPs and no study examined psychosocial consequences. This is somehow surprising as, in line with the transactional stress model, studies revealed associations between lower uncertainty intolerance and burnout or psychological distress in HCPs [10]. These associations might be strengthened by the high degree of uncertainties inherent in the context of rare diseases, necessitating the training of effective coping skills for HCPs. Further research is needed to explicitly evaluate psychological consequences of uncertainties experienced by HCPs in the context of rare diseases. Besides the effects for the HCPs themselves, parents might also benefit from an effective management of HCPs uncertainties when coping with their own burden, as they notice HCPs uncertainties [68]. Furthermore, more studies should define the term “uncertainty” more clearly and distinguish is from related constructs such as “worry” or “information needs”.

Although identified uncertainties are widely comparable to those experienced in other diseases [2], parents and HCPs share some unique uncertainties that are interrelated. This concerns scientific and practical uncertainties in particular. Unclear procedures of patient referral or unknown facilities to which patients should be referred to lead to uncertainties of caregivers about clinical responsibilities. HCPs’ uncertainties about rare disease symptoms and rare disease screening as well as unknown sources of information about rare diseases in general contribute to the diagnostic odyssey experienced by caregivers. Especially uncertainties related to information procurement are a topic that is unique to rare diseases. Interestingly only HCPs reported uncertainties concerning information procurement. Gathering information, as a strategy to cope with uncertainties might be used predominantly by parents resulting in knowledge about available sources of information [69]. Taken together, the uncertainties experienced by parental caregivers and HCPs frequently appear like two sides of the same coin.

## Funding

The study was conducted in the context of the professorship for health care research in rare diseases in children endowed by the Kindness-for-Kids Foundation. We acknowledge financial support from the Open Access Publication Fund of UKE - Universitätsklinikum Hamburg-Eppendorf.

## Data availability statement

All data accessed and analysed in this study are available in the article and its supplementary materials.

## CRediT authorship contribution statement

**David Zybarth:** Writing – review & editing, Writing – original draft, Visualization, Resources, Project administration, Methodology, Investigation, Formal analysis, Data curation, Conceptualization. **Laura Inhestern:** Writing – review & editing, Validation, Supervision, Resources, Methodology, Funding acquisition, Conceptualization. **Ramona Otto:** Writing – review & editing, Validation, Investigation, Data curation. **Corinna Bergelt:** Writing – review & editing, Supervision, Resources, Funding acquisition, Conceptualization.

## Declaration of competing interest

The authors declare that they have no known competing financial interests or personal relationships that could have appeared to influence the work reported in this paper.

## References

[1] Han P.K.J., Klein W.M.P., Arora N.K. (Nov. 2011). Varieties of uncertainty in health care: a conceptual taxonomy. Med. Decis. Making.

[2] Pomare C., Churruca K., Ellis L.A., Long J.C., Braithwaite J. (2019). A revised model of uncertainty in complex healthcare settings: a scoping review. J. Eval. Clin. Pract..

[3] Pope T.M. (2012). Legal fundamentals of surrogate decision making. Chest.

[4] Lazarus R.S., Folkman S. (1984).

[5] Schiffrin H.H., Nelson S.K. (Mar. 2010). Stressed and happy? Investigating the relationship between happiness and perceived stress. J. Happiness Stud..

[6] Cai R.Y., Uljarević M., Leekam S.R. (2020). Predicting mental health and psychological wellbeing in mothers of children with autism spectrum disorder: roles of intolerance of uncertainty and coping. Autism Res..

[7] Panjwani A.A., Millar B.M., Revenson T.A. (Feb. 2021). Tolerating uncertainty in the dark: insomnia symptoms, distress, and well-being among parents of adolescents and young adults with cancer. Int. J. Behav. Med..

[8] Bovier P.A., Perneger T.V. (2007). Stress from uncertainty from graduation to retirement—a population-based study of Swiss physicians. J. Gen. Intern. Med..

[9] Hancock J., Mattick K. (2020). Tolerance of ambiguity and psychological well-being in medical training: a systematic review. Med. Educ..

[10] Strout T.D. (2018). Tolerance of uncertainty: a systematic review of health and healthcare-related outcomes. Patient Educ. Counsel..

[11] Gerrity M.S., Earp J.A.L., DeVellis R.F., Light D.W. (1992). Uncertainty and professional work: perceptions of physicians in clinical practice. Am. J. Sociol..

[12] Lam J.H., Pickles K., Stanaway F.F., Bell K.J.L. (Dec. 2020). Why clinicians overtest: development of a thematic framework. BMC Health Serv. Res..

[13] Leigh S. (Mar. 2019). The cost of diagnostic uncertainty: a prospective economic analysis of febrile children attending an NHS emergency department. BMC Med..

[14] Andersen T. (Nov. 2012). The political empowerment of rare disease patient advocates both at EU and national level. Orphanet J. Rare Dis..

[15] Richter T. (Sep. 2015). Rare disease terminology and definitions-A systematic global review: report of the ISPOR rare disease special interest group. Value Health J. Int. Soc. Pharmacoeconomics Outcomes Res..

[16] Groft S.C., Posada de la Paz M., Posada de la Paz M., Taruscio D., Groft S.C. (2017). Advances in Experimental Medicine and Biology.

[17] Bauskis A., Strange C., Molster C., Fisher C. (Jun. 2022). The diagnostic odyssey: insights from parents of children living with an undiagnosed condition. Orphanet J. Rare Dis..

[18] Bogart K. (May 2022). Healthcare access, satisfaction, and health-related quality of life among children and adults with rare diseases. Orphanet J. Rare Dis..

[19] Benito-Lozano J. (Jul. 2023). Psychosocial impact at the time of a rare disease diagnosis. PLoS One.

[20] Dragojlovic N. (Feb. 2020). The cost trajectory of the diagnostic care pathway for children with suspected genetic disorders. Genet. Med..

[21] Rode J. (2005). Rare diseases: understanding this public health priority. EURORDIS Paris Fr.

[22] Mandic C.G., Johaningsmeir S., Corden T.E., Earle A., Acevedo-Garcia D., Gordon J.B. (Aug. 2017). Impact of caring for children with medical complexity on parents' employment and time. Community Work. Fam..

[23] Ziegler A., Antes G., König I. (Feb. 2011). Bevorzugte Report Items für systematische Übersichten und Meta-Analysen: Das PRISMA-Statement. DMW - Dtsch. Med. Wochenschr..

[24] Hong Q.N. (2018). Mixed methods appraisal tool (MMAT), version 2018. Regist. Copyr..

[25] Dixon-Woods M., Agarwal S., Jones D., Young B., Sutton A. (2005). Synthesising qualitative and quantitative evidence: a review of possible methods. J. Health Serv. Res. Policy.

[26] Azzopardi P.J. (Mar. 2020). Health-care providers' perspectives on uncertainty generated by variant forms of newborn screening targets. Genet. Med. Off. J. Am. Coll. Med. Genet..

[27] Zurynski Y. (2017). Rare disease: a national survey of paediatricians' experiences and needs. BMJ Paediatr. Open.

[28] Ramalle-Gómara E., Domínguez-Garrido E., Gómez-Eguílaz M., Marzo-Sola M.E., Ramón-Trapero J.L., Gil-de-Gómez J. (2020). Education and information needs for physicians about rare diseases in Spain. Orphanet J. Rare Dis..

[29] Vandeborne L., van Overbeeke E., Dooms M., De Beleyr B., Huys I. (2019). Information needs of physicians regarding the diagnosis of rare diseases: a questionnaire-based study in Belgium. Orphanet J. Rare Dis..

[30] Hamilton J.G. (2013). Sources of uncertainty and their association with medical decision making: exploring mechanisms in Fanconi anemia. Ann. Behav. Med..

[31] Kerr A.M., Haas S.M. (2014). Parental uncertainty in illness: managing uncertainty surrounding an" orphan" illness. J. Pediatr. Nurs..

[32] Kutsa O. (2022). Parental coping with uncertainties along the severe combined immunodeficiency journey. Orphanet J. Rare Dis..

[33] Lipinski S.E., Lipinski M.J., Biesecker L.G., Biesecker B.B. (2006). American Journal of Medical Genetics Part C: Seminars in Medical Genetics.

[34] Lundberg T., Lindström A., Roen K., Hegarty P. (2017). From knowing nothing to knowing what, how and now: parents' experiences of caring for their children with congenital adrenal hyperplasia. J. Pediatr. Psychol..

[35] Raspa M. (2020). Information and emotional support needs of families whose infant was diagnosed with SCID through newborn screening. Front. Immunol..

[36] Tluczek A., Chevalier McKechnie A., Lynam P.A. (2010). When the cystic fibrosis label does not fit: a modified uncertainty theory. Qual. Health Res..

[37] Whitmarsh I., Davis A.M., Skinner D., Bailey D.B. (2007). A place for genetic uncertainty: parents valuing an unknown in the meaning of disease. Soc. Sci. Med..

[38] de Ru M.H., Bouwman M.G., Wijburg F.A., van Zwieten M.C. (2012). Experiences of parents and patients with the timing of Mucopolysaccharidosis type I (MPS I) diagnoses and its relevance to the ethical debate on newborn screening. Mol. Genet. Metabol..

[39] Smits R.M. (Apr. 2022). Common needs in uncommon conditions: a qualitative study to explore the need for care in pediatric patients with rare diseases. Orphanet J. Rare Dis..

[40] Nguyen M.T., Charlebois K. (2015). The clinical utility of whole-exome sequencing in the context of rare diseases–the changing tides of medical practice. Clin. Genet..

[41] Anzelewicz S., Garnier H., Rangaswami A., Czauderna P. (Aug. 2017). Cultural, geographical and ethical questions when looking to enroll pediatric patients in rare disease clinical trials. Expert Opin. Orphan Drugs.

[42] Walkowiak D., Domaradzki J. (2021). Are rare diseases overlooked by medical education? Awareness of rare diseases among physicians in Poland: an explanatory study. Orphanet J. Rare Dis..

[43] Augustine E.F., Adams H.R., Mink J.W. (2013). Clinical trials in rare disease: challenges and opportunities. J. Child Neurol..

[44] Visibelli A., Roncaglia B., Spiga O., Santucci A. (Mar. 2023). The impact of artificial intelligence in the Odyssey of rare diseases. Biomedicines.

[45] Buck C., Doctor E., Hennrich J., Jöhnk J., Eymann T. (Jan. 2022). General practitioners' attitudes toward artificial intelligence–enabled systems: interview study. J. Med. Internet Res..

[46] Wojtara M., Rana E., Rahman T., Khanna P., Singh H. (2023). Artificial intelligence in rare disease diagnosis and treatment. Clin. Transl. Sci..

[47] Bragazzi N.L., Garbarino S. (Jun. 2024). Toward clinical generative AI: conceptual framework. JMIR AI.

[48] Pruniski B., Lisi E., Ali N. (2018). Newborn screening for Pompe disease: impact on families. J. Inherit. Metab. Dis..

[49] Inglese C.N. (2019). New developmental syndromes: understanding the family experience. J. Genet. Counsel..

[50] Qian Y., McGraw S., Henne J., Jarecki J., Hobby K., Yeh W.-S. (2015). Understanding the experiences and needs of individuals with spinal muscular atrophy and their parents: a qualitative study. BMC Neurol..

[51] van Scheppingen C., Lettinga A.T., Duipmans J.C., Maathuis K.G., Jonkman M.F. (2008). The main problems of parents of a child with epidermolysis bullosa. Qual. Health Res..

[52] Withers C.M. (Jan. 2021). Waiting for a diagnosis in Rubinstein-Taybi: the journey from “ignorance is bliss” to the value of “a label”. Am. J. Med. Genet. A..

[53] Kalbfell R., Wang W., Fishman S., Kerr A.M., Sisk B. (Jul. 2023). Burdens of disease and caregiver burden in complex vascular malformations. Pediatr. Blood Cancer.

[54] Xiao L. (Jul. 2023). Understanding caregiver experiences with disease-modifying therapies for spinal muscular atrophy: a qualitative study. Arch. Dis. Child..

[55] Khair K., Pelentsov L. (2019). Assessing the supportive care needs of parents with a child with a bleeding disorder using the Parental Needs Scale for Rare Diseases (PNS-RD): a single-centre pilot study. Haemophilia.

[56] Little T., Strodl E., Brown S., Mooney T. (2016). Parenting a child with haemophilia while living in a non-metropolitan area. J. Haemoph. Pract..

[57] Petersen A. (Jul. 2006). The best experts: the narratives of those who have a genetic condition. Soc. Sci. Med..

[58] Sandilands K., Williams A., Rylands A.J. (Dec. 2022). Carer burden in rare inherited diseases: a literature review and conceptual model. Orphanet J. Rare Dis..

[59] Inhestern L. (May 2024). Patient experiences of interprofessional collaboration and intersectoral communication in rare disease healthcare in Germany – a mixed-methods study. Orphanet J. Rare Dis..

[60] von der Lippe C., Diesen P.S., Feragen K.B. (Nov. 2017). Living with a rare disorder: a systematic review of the qualitative literature. Mol. Genet. Genomic Med..

[61] Inhestern L., Brandt M., Otto R., Zybarth D., Härter M., Bergelt C. (Aug. 2023). Versorgung von Menschen mit Seltenen Erkrankungen: Empfehlungen für eine gelungene intersektorale Zusammenarbeit. Bundesgesundheitsblatt - Gesundheitsforsch. - Gesundheitsschutz.

[62] Byrne N. (Aug. 2020). The role of primary care in management of rare diseases in Ireland. Ir. J. Med. Sci..

[63] Delisle V.C. (Jun. 2017). Perceived benefits and factors that influence the ability to establish and maintain patient support groups in rare diseases: a scoping review. Patient - Patient-Centered Outcomes Res.

[64] Pelentsov L.J., Fielder A.L., Laws T.A., Esterman A.J. (Jul. 2016). The supportive care needs of parents with a child with a rare disease: results of an online survey. BMC Fam. Pract..

[65] Obbarius N., Fischer F., Liegl G., Obbarius A., Rose M. (2021). A modified version of the transactional stress concept according to Lazarus and Folkman was confirmed in a psychosomatic inpatient sample. Front. Psychol..

[66] Hinton L., Armstrong N. (Aug. 2020). ‘“They don't know themselves, so how can they tell us?”: parents navigating uncertainty at the frontiers of neonatal surgery.’. Sociol. Health Illness.

[67] Faux D., Schoch K., Eubanks S., Hooper S.R., Shashi V. (2012). Assessment of parental disclosure of a 22q11. 2 deletion syndrome diagnosis and implications for clinicians. J. Genet. Counsel..

[68] Khangura S.D. (2016). Child and family experiences with inborn errors of metabolism: a qualitative interview study with representatives of patient groups. J. Inherit. Metab. Dis..

[69] Pelentsov L.J., Laws T.A., Esterman A.J. (Oct. 2015). The supportive care needs of parents caring for a child with a rare disease: a scoping review. Disabil. Health J.

