# Supplementary material for: Uncertainties of healthcare professionals and informal caregivers in rare diseases: A systematic review
Source: Heliyon. 2024 Sep 28;10(19):e38677. doi: 10.1016/j.heliyon.2024.e38677 (PMC11471567; doi:10.1016/j.heliyon.2024.e38677)
Supplement: Multimedia component 1 [file mmc1.docx]

*Risk of Bias Assessment - Mixed Methods Appraisal Tool (MMAT), version 2018*

| **Criteria for quantitative descriptive study designs** | | | | | |
| --- | --- | --- | --- | --- | --- |
| S1. Are there clear research questions? | | | | | |
| S2. Do the collected data allow to address the research questions? | | | | | |
| 4.1. Is the sampling strategy relevant to address the research question? | | | | | |
| 4.2. Is the sample representative of the target population? | | | | | |
| 4.3. Are the measurements appropriate? | | | | | |
| 4.4. Is the risk of nonresponse bias low? | | | | | |
| 4.5. Is the statistical analysis appropriate to answer the research question? | | | | | |
| **Criteria for qualitative study designs** | | | | | |
| S1. Are there clear research questions? | | | | | |
| S2. Do the collected data allow to address the research questions? | | | | | |
| 4.1. Is the sampling strategy relevant to address the research question? | | | | | |
| 4.2. Is the sample representative of the target population? | | | | | |
| 4.3. Are the measurements appropriate? | | | | | |
| 4.4. Is the risk of nonresponse bias low? | | | | | |
| 4.5. Is the statistical analysis appropriate to answer the research question? | | | | | |
| **possible answers** | | | | | |
|  | Yes |  | No |  | Can’t tell/ Criteria partly fulfilled |

*Healthcare Professionals*

| Azzopardi, Upshur et al. (2020) | S1 | S2 | 1.1 | 1.2 | 1.3 | 1.4 | 1.5 |
| --- | --- | --- | --- | --- | --- | --- | --- |
| Nguyen and Charlebois (2015) | S1 | S2 | 1.1 | 1.2 | 1.3 | 1.4 | 1.5 |
| Ramalle-Gómara, Domínguez-Garrido et al. (2020) | S1 | S2 | 4.1 | 4.2 | 4.3 | 4.4 | 4.5 |
| Vandeborne, van Overbeeke et al. (2019) | S1 | S2 | 4.1 | 4.2 | 4.3 | 4.4 | 4.5 |
| Zurynski, Gonzalez et al. (2017) | S1 | S2 | 4.1 | 4.2 | 4.3 | 4.4 | 4.5 |

*Caregiver*

| de Ru, Bouwman et al. (2012) | S1 | S2 | 1.1 | 1.2 | 1.3 | 1.4 | 1.5 |
| --- | --- | --- | --- | --- | --- | --- | --- |
| Faux, Schoch et al. (2012) | S1 | S2 | 1.1 | 1.2 | 1.3 | 1.4 | 1.5 |
| Hamilton, Hutson et al. (2013) | S1 | S2 | 4.1 | 4.2 | 4.3 | 4.4 | 4.5 |
| Hinton and Armstrong (2020) | S1 | S2 | 1.1 | 1.2 | 1.3 | 1.4 | 1.5 |
| Inglese, Elliott et al. (2019) | S1 | S2 | 1.1 | 1.2 | 1.3 | 1.4 | 1.5 |
| Kalbfell, Wang et al. (2023) | S1 | S2 | 1.1 | 1.2 | 1.3 | 1.4 | 1.5 |
| Kerr and Haas (2014) | S1 | S2 | 1.1 | 1.2 | 1.3 | 1.4 | 1.5 |
| Kutsa, Andrews et al. (2022) | S1 | S2 | 1.1 | 1.2 | 1.3 | 1.4 | 1.5 |
| Lipinski, Lipinski et al. (2006) | S1 | S2 | 4.1 | 4.2 | 4.3 | 4.4 | 4.5 |
| Lundberg, Lindström et al. (2017) | S1 | S2 | 1.1 | 1.2 | 1.3 | 1.4 | 1.5 |
| Pruniski, Lisi and Ali (2018) | S1 | S2 | 1.1 | 1.2 | 1.3 | 1.4 | 1.5 |
| Qian, McGraw et al. (2015) | S1 | S2 | 1.1 | 1.2 | 1.3 | 1.4 | 1.5 |
| Raspa, Kutsa et al. (2023) | S1 | S2 | 1.1 | 1.2 | 1.3 | 1.4 | 1.5 |
| Smits, Vissers et al. (2022) | S1 | S2 | 1.1 | 1.2 | 1.3 | 1.4 | 1.5 |
| Tluczek, McKechnie and Lynam (2010) | S1 | S2 | 1.1 | 1.2 | 1.3 | 1.4 | 1.5 |
| van Scheppingen, Lettinga et al. (2008) | S1 | S2 | 1.1 | 1.2 | 1.3 | 1.4 | 1.5 |
| Whitmarsh, Davis et al. (2007) | S1 | S2 | 1.1 | 1.2 | 1.3 | 1.4 | 1.5 |
| Withers, Fleming et al. (2020) | S1 | S2 | 1.1 | 1.2 | 1.3 | 1.4 | 1.5 |
| Xiao, Kang et al. (2023) | S1 | S2 | 1.1 | 1.2 | 1.3 | 1.4 | 1.5 |
