# Supplementary material for: Uncertainties of healthcare professionals and informal caregivers in rare diseases: A systematic review
Source: Heliyon. 2024 Sep 28;10(19):e38677. doi: 10.1016/j.heliyon.2024.e38677 (PMC11471567; doi:10.1016/j.heliyon.2024.e38677)
Supplement: Multimedia component 2 [file mmc2.docx]

*Search Strategy*

*Pubmed (incl. MEDLINE):*

**((uncertainty[MeSH Terms]) OR (decision making[MeSH Terms])) OR (((((((((uncertain) OR (uncertain*)) OR (certain*)) OR (unsure)) OR (ambig*)) OR (decision making)) OR (clinical decision)) OR (ignorance)))** AND

((rare disease[MeSH Terms]) OR (neonatal screening[MeSH Terms])) OR ((((((((((((((((((((((((((((((((((((((((((Rare Transplant-Related Diseases) OR (Rare Systemic And Rheumatological Diseases Of Childhood)) OR (Chromosomal Anomalies Sorted By Chromosome)) OR (Rare Teratologic Disorders)) OR (Rare Allergic Disease)) OR (Rare Surgical Maxillofacial Diseases)) OR (Rare Gynaecological And Obstetric Diseases)) OR (Rare Diseases Due To Toxic Effects)) OR (Rare Infectious Diseases)) OR (Rare Neoplastic Diseases)) OR (Rare Infertility)) OR (Rare Otorhinolaryngological Diseases)) OR (Rare Bone Diseases)) OR (Rare Circulatory System Diseases)) OR (Rare Odontological Diseases)) OR (Rare Systemic And Rheumatological Diseases)) OR (Rare Immunological Diseases)) OR (Rare Haematological Diseases)) OR (Rare Endocrine Diseases)) OR (Rare Ophthalmic Diseases)) OR (Rare Renal Diseases)) OR (Rare Skin Diseases)) OR (Rare Surgical Thoracic Diseases)) OR (Rare Urogenital Diseases)) OR (Rare Respiratory Diseases)) OR (Rare Hepatic Diseases)) OR (Rare Abdominal Surgical Diseases)) OR (Rare Neurological Diseases)) OR (Rare Genetic Diseases)) OR (Rare Gastroenterological Diseases)) OR (Rare Inborn Errors Of Metabolism)) OR (Rare Sucking Swallowing Diseases)) OR (Rare Cardiac Malformations)) OR (Rare Developmental Anomalies During Embryogenesis)) OR (Rare Cardiac Diseases)) OR (rare disease)) OR (orphan disease)) OR (rare disorder)) OR (orphan disorder) OR (newborn screening) OR (neonatal screening))))) AND

(health personnel[MeSH Terms]) OR ((((((((((((((((((((((healthcare provider) OR (healthcare professional)) OR (health care provider)) OR (health care professional)) OR (pediatric*)) OR (physician*)) OR (clinician*)) OR (general practitioner)) OR (medical resident)) OR (nurse)) OR (caregiver)) OR (caregiv*)) OR (carer)) OR (mother)) OR (father)) OR (parent)) OR (family)) OR (mother*)) OR (father*)) OR (parent*)) OR (famil*)))

*OVID (APA PsycArticles, APA PsycInfo, PSYNDEXplus Databases):*

#1 (healthcare provider or health care provider or healthcare professional or health care professional or pediatric* or physician* or clinician* or general practitioner or medical resident or nurse or caregiv* or carer or mother* or father* or parent* or famil*).af.

#2 health personnel.mh.

#3 1 OR 2

#4 (Rare Transplant-Related Diseases or (Rare Systemic and Rheumatological Diseases Of Childhood) or Chromosomal Anomalies Sorted By Chromosome or Rare Teratologic Disorders or Rare Allergic Disease or Rare Surgical Maxillofacial Diseases or (Rare Gynaecological and Obstetric Diseases) or Rare Diseases Due To Toxic Effects or Rare Infectious Diseases or Rare Neoplastic Diseases or Rare Infertility or Rare Otorhinolaryngological Diseases or Rare Bone Diseases or Rare Circulatory System Diseases or Rare Odontological Diseases or (Rare Systemic and Rheumatological Diseases) or Rare Immunological Diseases or Rare Haematological Diseases or Rare Endocrine Diseases or Rare Ophthalmic Diseases or Rare Renal Diseases or Rare Skin Diseases or Rare Surgical Thoracic Diseases or Rare Urogenital Diseases or Rare Respiratory Diseases or Rare Hepatic Diseases or Rare Abdominal Surgical Diseases or Rare Neurological Diseases or Rare Genetic Diseases or Rare Gastroenterological Diseases or Rare Inborn Errors Of Metabolism or Rare Sucking Swallowing Diseases or Rare Cardiac Malformations or Rare Developmental Anomalies During Embryogenesis or Rare Cardiac Diseases or rare disease or orphan disease or rare disorder or orphan disorder or newborn screening or neonatal screening).af.

#5 (rare diseases or neonatal screening).mh.

#6 4 OR 5

#7 (uncertain* or certain* or ambig* or unsure or ignorance or decision making or clinical decision).af.

#8 (uncertainty or decision making).mh.

#9 7 OR 8

#10 3 AND 6 AND 9

*CINAHL:*

#1 TX (healthcare provider or health care provider or healthcare professional or health care professional or pediatric* or physician* or clinician* or general practitioner or medical resident or nurse or caregiv* or carer or mother* or father* or parent* or famil*)

#2 SU (health personnel)

#3 S1 OR S2

#4 SU (decision making or uncertainty)

#5 TX (uncertain* or certain* or ambig* or unsure or ignorance or decision making or clinical decision)

#6 S4 OR S5

#7 SU (rare diseases or neonatal screening)

#8 TX (Rare Transplant-Related Diseases or (Rare Systemic and Rheumatological Diseases Of Childhood) or Chromosomal Anomalies Sorted By Chromosome or Rare Teratologic Disorders or Rare Allergic Disease or Rare Surgical Maxillofacial Diseases or (Rare Gynaecological and Obstetric Diseases) or Rare Diseases Due To Toxic Effects or Rare Infectious Diseases or Rare Neoplastic Diseases or Rare Infertility or Rare Otorhinolaryngological Diseases or Rare Bone Diseases or Rare Circulatory System Diseases or Rare Odontological Diseases or (Rare Systemic and Rheumatological Diseases) or Rare Immunological Diseases or Rare Haematological Diseases or Rare Endocrine Diseases or Rare Ophthalmic Diseases or Rare Renal Diseases or Rare Skin Diseases or Rare Surgical Thoracic Diseases or Rare Urogenital Diseases or Rare Respiratory Diseases or Rare Hepatic Diseases or Rare Abdominal Surgical Diseases or Rare Neurological Diseases or Rare Genetic Diseases or Rare Gastroenterological Diseases or Rare Inborn Errors Of Metabolism or Rare Sucking Swallowing Diseases or Rare Cardiac Malformations or Rare Developmental Anomalies During Embryogenesis or Rare Cardiac Diseases or rare disease or orphan disease or rare disorder or orphan disorder or newborn screening or neonatal screening)

#9 S7 OR S8

#10 S3 AND S6 AND S9
